# Fabrication and Mechanical Testing of the Uniaxial Graded Auxetic Damper

**DOI:** 10.3390/ma15010387

**Published:** 2022-01-05

**Authors:** Hasan Al-Rifaie, Nejc Novak, Matej Vesenjak, Zoran Ren, Wojciech Sumelka

**Affiliations:** 1 Faculty of Civil Engineering and Transport, Poznan University of Technology, 60-965 Poznan, Poland; wojciech.sumelka@put.poznan.pl; 2 Faculty of Mechanical Engineering, University of Maribor, 2000 Maribor, Slovenia; n.novak@um.si (N.N.); matej.vesenjak@um.si (M.V.); zoran.ren@um.si (Z.R.)

**Keywords:** uniaxial graded auxetic damper, energy absorber, mechanical properties, finite element method, explicit solver

## Abstract

Auxetic structures can be used as protective sacrificial solutions for impact protection with lightweight and excellent energy-dissipation characteristics. A recently published and patented shock-absorbing system, namely, Uniaxial Graded Auxetic Damper (UGAD), proved its efficiency through comprehensive analytical and computational analyses. However, the authors highlighted the necessity for experimental testing of this new damper. Hence, this paper aimed to fabricate the UGAD using a cost-effective method and determine its load–deformation properties and energy-absorption potential experimentally and computationally. The geometry of the UGAD, fabrication technique, experimental setup, and computational model are presented. A series of dog-bone samples were tested to determine the exact properties of aluminium alloy (AW-5754, T-111). A simplified (elastic, plastic with strain hardening) material model was proposed and validated for use in future computational simulations. Results showed that deformation pattern, progressive collapse, and force–displacement relationships of the manufactured UGAD are in excellent agreement with the computational predictions, thus validating the proposed computational and material models.

## 1. Introduction

External dampers (or energy absorbers) are necessary for critical structures which do not have suitable damping facilities to absorb dynamic loads. Special attention is needed for dampers (thought as protective systems), which have applications in mechanical, civil, and aerospace engineering, e.g., as seismic vibration controllers in multi-story structures [1]. Among many possible classifications, dampers can be active or passive in the sense that external power is or is not necessary, respectively. Herein, when impact/blast absorbers are considered, they should be passive, as a power cut is highly expected. One should notice that, e.g., earthquake dampers (such as fluid viscous dampers) might not be suitable for such applications as for large-strain-impact dynamics as they generally need a longer time to respond. Therefore, when blast waves are to be damped, absorption through plastic deformation is a suitable alternative (such as plastic deformation of cellular materials/structures).

Cellular materials (CM) are one of the most promising materials for modern high-end engineering applications. CM should be thought of as materials with internal cellular structure either on nano-, micro-, meso-, or macro-scale. Herein, many different base materials can be considered with an internal cellular structure of different morphologies, which results in unique and attractive mechanical and thermal properties [2,3]. Different cellular structures have been investigated, considering their critical aspects vs. their applications in engineering, medicine, and other fields [4,5]. One can say that, due to their positive attributes such as light weight, high energy-absorption capacity, high damping efficiency, and/or thermal insulation, they are one of the most promising materials for future applications [6,7]. Although some CM are already being used in practical applications [8], their common use in real-life applications is limited because most of them are still on conceptual level [9,10,11,12]. What is more, mass-production technologies often lack control of cell pores’ shape, size, and distribution during the fabrication process, which results in scattering of their mechanical properties. Due to these reasons, unidirectional structures, such as Lotus [11], UniPore [12], metallic hollow sphere structures [13], and advanced pore morphology foam [14,15,16], have recently been proposed to overcome those shortcomings. For interested readers, selected manufacturing methods of CM are described in [5,17]. 

Modern CM with constant or spatially graded porosity distribution needs special and advanced additive manufacturing technologies (AMT) for practical purposes. AMT provides additionally the possibility that the cellular structure exhibits a negative Poisson’s ratio under mechanical loading. Such structures expand in the lateral direction when subjected to longitudinal tensile loading and show lateral contraction in the case of longitudinal compressive loading [18]. This behaviour comes from a properly designed (2D or 3D) hinge- or sheet-like cellular skeleton with predesigned geometry [19,20]. One calls such CM auxetic cellular metamaterials (ACM). Among others, ACM offers extraordinary mechanical properties, i.e., unique deformation behaviour, variable stiffness, density, significant mechanical energy absorption through deformation, non-intuitive bending behaviour, and increased shear resistance. Moreover, ACM reveals noticeable volume changes during loading, which is crucial for applications such as crash absorbers, stents, and protective padding [21]. The ACM cells also deform rapidly through the entire cellular structure under loading, and therefore they are effective for spreading the loading to the structure. Hence, in the case of an impact on ACM, the energy dissipates through its entire structure. Among three main groups of ACM, i.e., auxetic honeycombs, auxetic microporous polymers, and auxetic composites, in this research the auxetic honeycomb structure with re-entrant topology was chosen, which provides optimal performance compared to other auxetic topologies [22].

Recently, experimental studies of ACM covered mainly the deformation of ‘uniform’ auxetic structures under dynamic/quasi-static loading at large strains, small strains [23], or even bending [24]. Hence, to the authors’ knowledge, limited studies have considered the uniaxial quasi-static compression tests of ‘graded’ auxetic structures. The situation is even more incomplete for experimental studies that investigated the dynamic response of ACM (especially for different strain rates and scales). Furthermore, they primarily focused on structures of the same geometry, porosity, and direction of loading or strain rate in a particular experiment [22,25,26,27,28,29].

Nowadays, productions of new ACM need designing on the level of computational simulations. Those methods are usually based on the finite element method (FEM), which gives a deep understanding of their behaviours, i.e., vital for evaluating the deformation behaviour and mechanical response of various cellular materials and their potential applications. Herein, different modelling techniques can be used, i.e., the computational models can be based on homogenisation theory [30], beam theories [31], shell [32], and volume theories [33], depending on the desired precision of geometry. However, independently of a chosen numerical technique, they always need to be validated to achieve a good correlation between experimental and computational results. The numerical models are generally used to optimise the internal cellular structure to obtain the desired response of the component or material [34]. The final quality of computational results can be as close as 85 to 95% compared to experiments. 

Time-dependent numerical studies were also conducted to analyse the response of auxetic structures (as a sacrificial energy absorbers) under impact [35,36], blast [37], or ballistic scenarios [38]. 

The previous research of Al-Rifaie et al. [39] aimed to design, utilising numerical modelling tools, a new damper that can be used as a shock/blast energy absorber for different structural applications (such as improving the blast-resistance of building’s façade or doors and absorbing unexpected crashes of elevators or motor vehicles’ front bumpers). A thorough parametric study was conducted to achieve the most efficient graded auxetic system. The proposed damper was numerically tested and validated analytically. They concluded that using three auxetic cores (with different cell-wall thicknesses) gave a strength range from 1 to 10 MPa and a wider plateau region (80% of total crushing strain) that can justify the excellent performance of the UGAD under different blast intensities. The authors pointed out that the lack of experimental validation represents a limitation of their work and urged further experimental validation. Hence, the current research aimed to fabricate and test the UGAD experimentally and numerically. It is worth mentioning that the UGAD idea was recently patented by the patent office of the Republic of Poland [40]. Moreover, the blast/impact protection potential of the UGAD on the structural level was assessed numerically and published in [7]. 

In this study, the manufacturing of the UGAD consisted of a relatively cheap modified fabrication technique, inspired by Remennikov et al. [41]. They manufactured large-scale re-entrant auxetic panels by corrugating aluminium sheets into the desired geometry/topology and gluing them using low-functionality polyurethane-based structural adhesive. Remennikov et al. [41] found that the chosen adhesive could not fill the gaps appropriately and could not adequately bond the two sheets. Hence, they used an additional three lines of 3.2 mm pop rivets to connect the corrugated profiles. In the current research, smaller auxetic units were produced to fit into the UGAD damper body. Moreover, a different aluminium alloy was used in addition to a stronger glue/adhesive that does not require extra rivets. This consequently increased their mechanical performance and reduced the production time and cost. The research also covered the development of a nonlinear finite element model of the UGAD with an experimentally validated material model that can be used to customise, analyse, and predict the response for further applications. 

In short, this study aimed to fabricate and test experimentally (for the first time) a recently patented concept of a Uniaxial Graded Auxetic Damper (UGAD). The main four objectives of this research were: proposing a simplified material model of the tested aluminium alloy (that shows better convergence and more-stable computational simulations); fabricating the UGAD using a non-expensive technique rather than 3D printing; experimental testing of three UGAD samples under quasi-static loading; and building a verified nonlinear finite element model for future applications. The UGAD is composed of three auxetic cores, a damper body, and a piston. The three auxetic cores have three different cell-wall thickness (t) values to achieve a graded auxetic damper that can absorb low, medium, and high energy, as approved numerically in an earlier investigation [7,39]. The four objectives listed above summarise the novelty in this research. 

## 2. Fabrication and Experimental Testing

### 2.1. Geometry and Fabrication of the UGAD

**Figure 1:**
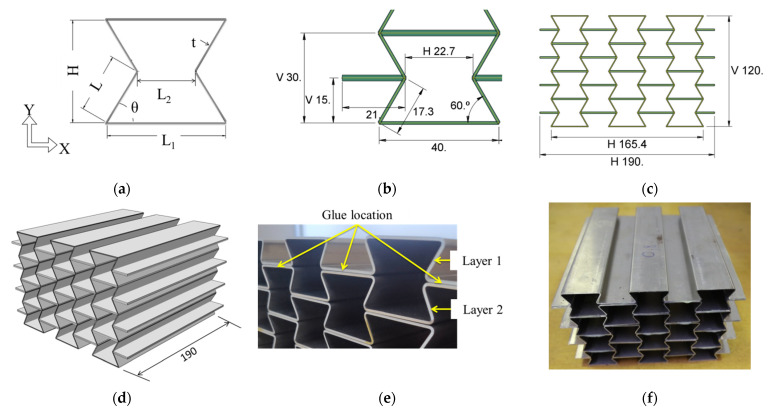
The geometry and fabrication procedure of the auxetic core. (**a**) The nomenclature for the auxetic cell; (**b**) the dimensions of the auxetic cell; (**c**) the cross-sectional dimensions of the auxetic core; (**d**) the extrusion depth of the auxetic core; (**e**) the corrugated aluminium layers and glue locations; (**f**) the glued layers, forming an auxetic core.

The geometry of the manufactured auxetic cores was adopted from a detailed parametric study of Al-Rifaie and Sumelka [39] and is presented in Figure 1. Each cell has a width (L1) of 40 mm and a height (H) of 30 mm with L = 17.3 mm (Figure 1a,b). Hence, one auxetic core of 5 × 4 cells has a total width of 190 mm, a height of 120 mm, and a depth of 190 mm (Figure 1c,d). According to [42], increasing the number of cells showed an exponential rise in Poisson’s ratio and improved the auxetic properties of the re-entrant structure. However, to keep the UGAD concept as portable as possible, four cells were selected for each auxetic core (Figure 1c).

The fabrication procedure was adopted from Remennikov et al. [41], who first manufactured large-scale auxetic panels. The auxetic cores were fabricated from corrugated aluminium sheets made of aluminium alloy (AW-5754, T111, density $\rho_{s}$ = 2660 kg/m^3^, Impol, SLOVENSKA BISTRICA, Slovenia), formed to the specified geometry, that were glued together with epoxy adhesive *LOCTITE^®^ EA 9466* (DÜSSELDORF, Germany), as in Figure 1e,f. The adhesive cures at room temperature, creating a strong bond with good peel resistance and shear strength. It is designed for applications where a long lifetime and strong bond strength are required.

**Figure 2:**
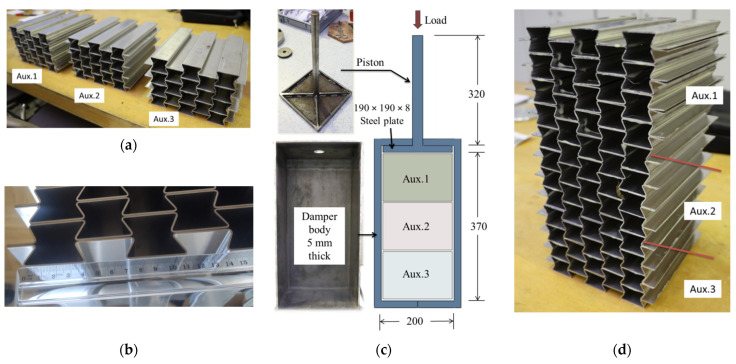
Dimensions and components of the UGAD. (**a**) The three manufactured auxetic cores that have different cell-wall thicknesses; (**b**) the accuracy of the corrugations; (**c**) the cross-sectional dimensions of the UGAD with its components (piston and damper body); (**d**) the three auxetic cores placed on top of each other.

The dimensions and components of the fabricated UGAD are shown in Figure 2. The UGAD is composed of three auxetic cores, damper body, and piston. Three different auxetic cores (namely, Aux.1, Aux.2, and Aux.3) were used with three different cell-wall thickness (t) values (selected here as 0.8, 1.0, and 1.2 mm) to achieve a graded auxetic damper. Aux.1 (with the smallest t) was placed on the top, and Aux.2 was in the middle, while Aux.3 was at the bottom. The cores Aux.1, Aux.2, and Aux.3 are dedicated to absorbing low-, medium-, and high-impact energy. In such a manner, a progressive collapse is achieved, and the UGAD would have three different dynamic crushing strengths, as shown in Figure 2a,d. Figure 2b shows the fabrication accuracy of the corrugated layers of an auxetic core. It is essential to mention that, although layers of a single auxetic core are glued together (Figure 2a), the contacting surfaces between the auxetic cores are not glued (Figure 2d). This allows for easier replacement of the crushed core in UGAD after an impact event without the need to replace all three cores. The damper body has 370 mm × 200 mm × 200 mm internal space and a wall thickness of 8 mm (Figure 2c). The piston has a 20 mm circular rod welded to 190 mm ×190 mm steel plate with cross-shape stiffeners (Figure 2c). The damper body and piston are made of structural steel S235 (JR, Štore Steel, ŠTORE, Slovenia).

The properties of the three auxetic cores are listed in Table 1. All have the same L, cell angle θ = 60°, grade, density, size and, therefore, overall volume. Different cell-wall thickness (t) leads to changes in mass, density, relative density, and porosity of the three studied auxetic cores. The actual density of each auxetic core (ρ) was calculated by dividing the mass (of the core) by the undeformed volume V (V = 190 mm × 190 mm × 120 mm = 0.004332 m^3^). The relative density $\rho^{*}$ is the ratio of the auxetic core density (ρ) and the density of the solid base material ($\rho_{s}$):

(1) $$\rho^{*}=\rho /\rho_{s}$$

The relative density $\rho^{*}$ can also be found analytically using [4]:

(2) $$\rho^{*}=\frac{\rho }{\rho_{s}}=\frac{1}{2}\;\frac{t}{L}\;\frac{\left(\frac{L_{1}}{L}+2\right)}{\cos\theta \left(\frac{L_{1}}{L}+\sin\theta \right)}\;$$

From the relative density, the porosity can be calculated as:

(3) $$p=100(1-\rho^{*})$$

From Table 1, it can be perceived that the three auxetic cores are generally lightweight with low relative density (high porosity). The relative density increased with increasing (*t*) while the porosity decreased. Aux.1 had the highest porosity of 90.4% compared to Aux.3 with 86.1%.

### 2.2. Material Properties of the Aluminium Alloy

**Figure 3:**
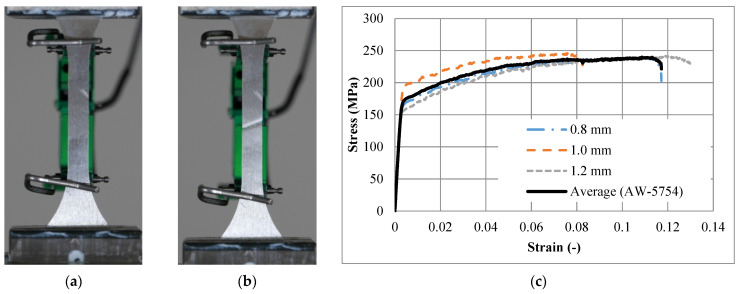
Tensile testing of the aluminium alloy (AW-5754, T111): (**a**) loading a dog-bone sample of thickness 1.0 mm; (**b**) dog-bone sample of thickness 1.0 mm after failure; (**c**) the stress–strain relationship of the three sets (for three different thicknesses) and the average (representing the tested aluminium alloy).

A series of standard tensile tests were conducted on the universal testing machine INSTRON^®^ 8801(NORWOOD, Massachusetts, USA) at the constant loading velocity of 0.1 mm/s to determine the exact mechanical properties of the used aluminium alloy (AW-5754, T111, Impol, SLOVENSKA BISTRICA, Slovenia). A total of 12 dog-bone samples were tested according to the DIN50125 standard, using a clip-on extensometer with initial length of 50 mm. They were categorised into three sets, namely, first, second and third, with thicknesses of 0.8 mm, 1.0 mm, and 1.2 mm, respectively. The average behaviour of each group was considered, and the corresponding mechanical properties were calculated. Figure 3a,b show the dog-bone sample of thickness 1.0 mm, before and after failure, respectively. Figure 3c shows the derived engineering stress–strain relationships for all three sets (for three different thicknesses) and the average response (black line). To see the scatter of the experimental tensile test results compared to the mean, the standard deviation was calculated for each strain value. Then, the maximum and average standard deviation values were calculated as 19.58 and 12.05, respectively.

**Figure 4:**
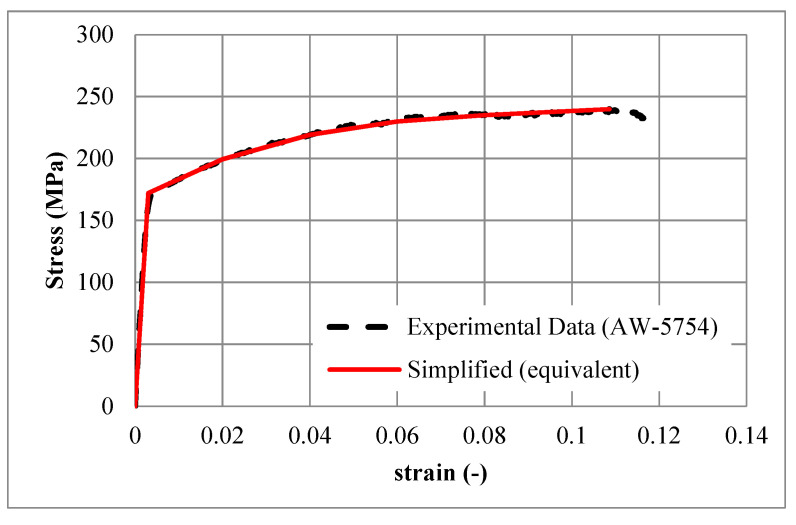
The experimental (lab. based) and simplified (equivalent) stress–strain relationship of the aluminium alloy (AW-5754, T-111).

Young’s modulus was calculated as 63,177 MPa. The Poisson’s ratio of 0.33 was assumed from the literature [43]. An equivalent simplified relationship is proposed to simplify the actual stress–strain relationship for the purpose of better convergence and more-stable computational simulations. Table 2 lists the key points of the equivalent simplified stress–strain relationship used for the material (constitutive) model. Figure 4 compares the measured (experiment) and simplified (equivalent) stress–strain relationship of the (AW-5754, T-111) aluminium.

### 2.3. Experimental Testing of the UGAD

**Figure 5:**
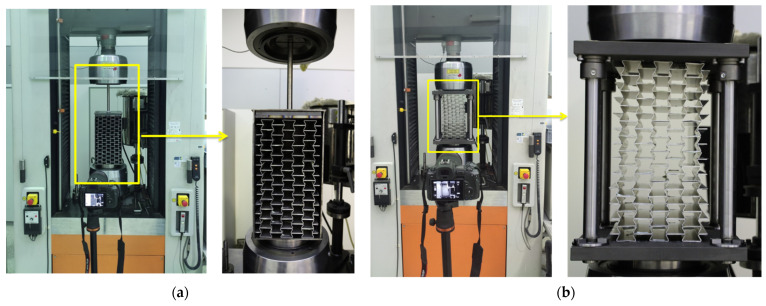
Experimental testing using a quasi-static compression testing machine: (**a**) testing the whole UGAD, including the damper body and the piston; (**b**) testing the three auxetic cores.

The experimental testing was performed using an INSTRON^®^ 1225 quasi-static compression testing machine (NORWOOD, Massachusetts, USA) with a maximum capacity of 250 kN. The loading velocity was set to 0.5 mm/s. Figure 5a shows the loading of the manufactured UGAD, including the damper body and the piston. However, to ensure a valid comparison with the computational model (presented in the next section) and exclude possible behaviour uncertainties from the damper body/piston, it was decided to test only the three auxetic cores (Figure 5b). The results (in Section 4) show the behaviour of the UGAD core and justify the reason for such an experimental approach. To determine behaviour discrepancy and calculate the average response, three similar sets were tested (namely, UGAD 1, UGAD 2, and UGAD 3), each containing the three auxetic cores (Aux.1, Aux.2, and Aux.3) defined earlier in Table 1. 

## 3. Computational Simulations

### 3.1. Computational Model

**Figure 6:**
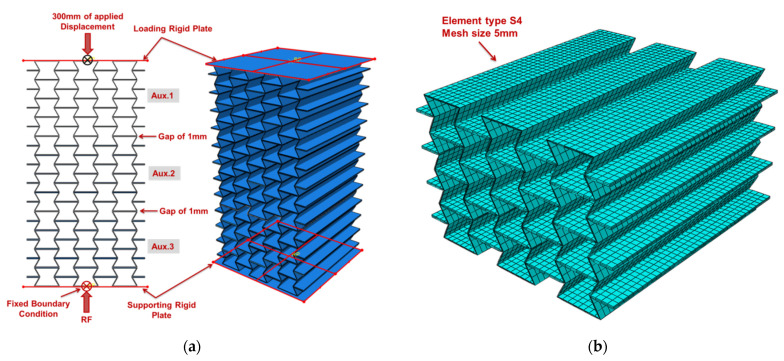
The computational model of the UGAD: (**a**) the side and 3D views with boundary conditions; (**b**) finite element mesh of one auxetic core.

The three auxetic cores of the UGAD were modelled using Abaqus/CAE (Version 2019, Dassault Systèmes, Vélizy-Villacoublay, France) and analysed using the Explicit solver (Version 2019, Dassault Systèmes, Vélizy-Villacoublay, France). A homogeneous isotropic section was defined using the selected aluminium alloy (Section 2.2). The material model was assumed to be elastic-plastic with strain-hardening properties, based on the mechanical properties presented in Table 2. A loading plate and a supporting plate (200 mm × 200 mm × 1 mm, each) were located above and under the three cores to replicate the experimental testing scheme. The two plates were assigned as rigid bodies to reduce the computational time. The explicit general contact was defined to model interactions between the assembled parts. The interaction property assumed a “hard” normal behaviour and a “penalty” friction formulation with a coefficient of friction of 0.3. An initial gap of 1 mm was prescribed between the three cores in the model (Figure 6a) to simulate that the cores are not welded/tied/connected. The mesh consisted of linear S4 shell elements (four-node doubly curved element) with five points of integration through the thickness. The mesh convergence analysis revealed that a mesh size of 5 mm was the most appropriate for cost-efficient computational simulations. 

### 3.2. Loading

While replicating the experimental quasi-static loading, the computational analyses could not converge using the Static/Abaqus-standard solver since the computational model contained several nonlinearities (plasticity and contacts between the aluminium plates). Hence, the Abaqus/Explicit dynamic solver was applied, which is well-observed in literature and approved by the software documentation [44]. According to Abaqus documentations, “The explicit dynamics procedure is typically used to solve two classes of problems: transient dynamic response calculations and quasi-static simulations involving complex nonlinear effects (most commonly problems involving complex contact conditions)”. The time frame for the explicit simulation was set to 0.15 s to reduce the computational time. The actual time scale is generally not important for quasi-static simulations incorporating rate-independent material behaviour [45]. The loading was applied on the reference point of the top compressing rigid plate as a predefined displacement of 300 mm downward. The displacement followed a linear pattern starting from zero at t = 0 s, and 300 mm at t = 0.15 s. The reaction force is theoretically equivalent to the load required to cause such displacement/compression at the reference point of the bottom supporting rigid plate. Other parameters of interest are stress, deformation, and strain energy.

## 4. Results and Discussion

**Figure 7:**
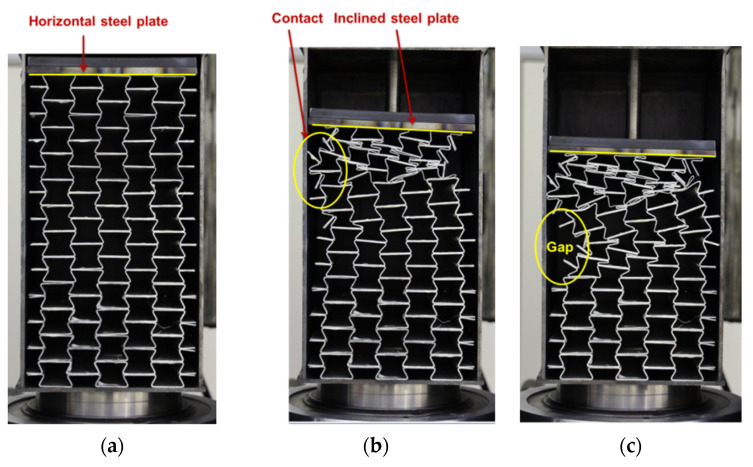
Experimental testing of the whole UGAD: (**a**) unloaded (at zero displacement); (**b**) loaded (at 65 mm displacement); (**c**) loaded (at 95 mm displacement).

The experimental testing of the UGAD, including the damper body and the piston, led to non-desirable results (Figure 7). The piston’s compressing steel plate was horizontal when the UGAD was unloaded (at zero displacements). When loaded, the plate started to incline at different angles based on the deformation/collapse of the auxetic unit cells. As a result, the deformation pattern of the auxetic cores changed. Figure 7b presents the lateral shift of Aux.1 to the left, causing unexpected lateral force on the damper body. Moreover, at a displacement of 65 mm, Aux.2 started to show an unsymmetrical gap and collapse on one side. This poor performance of the UGAD was due to the non-stiffened joint between the piston rod and the piston’s plate (Figure 2c). For future research, the authors suggest using extra stiffeners to solve the problem mentioned above. Hence, it was decided to test only the three auxetic cores separately and compare them with a corresponding computational model. 

**Figure 8:**
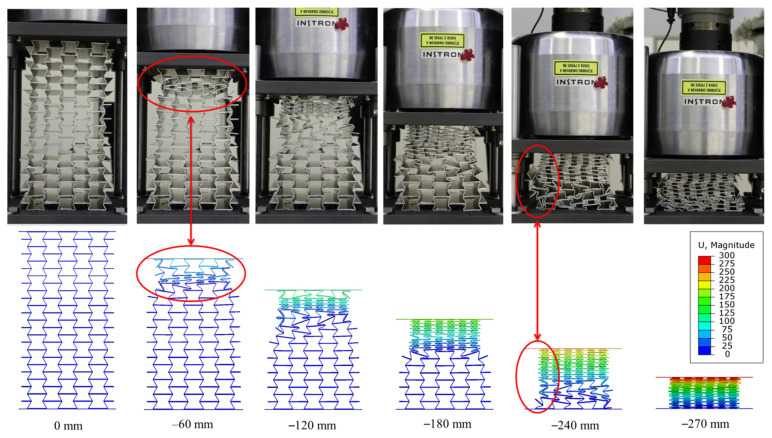
The deformation of the UGAD at different displacements, comparing experimental (UGAD1) and numerical results.

Figure 8 presents the behaviour of the auxetic cores (e.g., UGAD 1) under different applied displacements, and compares the experimental results with the computational model. The experimentally observed progressive collapse of the manufactured auxetic cores is in good agreement with the computational prediction. At 60 mm of applied displacement, the negative Poisson’s ratio is visible with consistent and stable auxetic performance. A slight lateral buckling (deviation from the computational model) appeared when Aux.3 (with t = 1.2 mm) was being compacted (at 240 mm of applied displacement). The lateral buckling of Aux.3 can be linked to the higher compressing force. The epoxy adhesive was successfully applied for bonding the auxetic layers with high efficiency.

**Figure 9:**
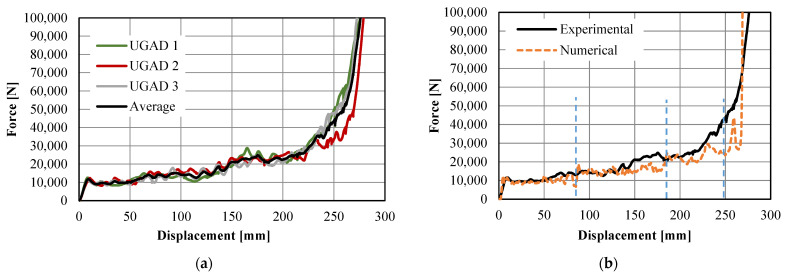
The force–displacement curves of the UGAD: (**a**) experimental results; (**b**) experimental vs. numerical results.

The force–displacement relationships of the UGADs (UGAD 1, UGAD 2, and UGAD 3), with their average, are presented in Figure 9a. The three tests showed high similarity, with a relatively small deviation. Figure 9b shows the comparison of the average experimental force–displacement response with the computational results. The force rises linearly to 11,000 N, followed by three step-wise plateau zones ending with a densification zone at displacements >250 mm. The first plateau zone (Aux.1) is at approx. 10 kN between 4 and 85 mm of applied displacement. The second plateau zone (Aux.2) is at approx. 15 kN between 85 and 185 mm of applied displacement. The third plateau zone (Aux.3) sharply fluctuates between 20 and 40 kN and between 185 and 250 mm of applied displacement. The slight difference between the experimental and computational relationship appears mainly at higher loads due to possible local damage, extensive friction, and lateral buckling perceived in the fabricated auxetic cores. In general, the experimental and computational results are in good agreement, which validates the used computational and material models.

**Figure 10:**
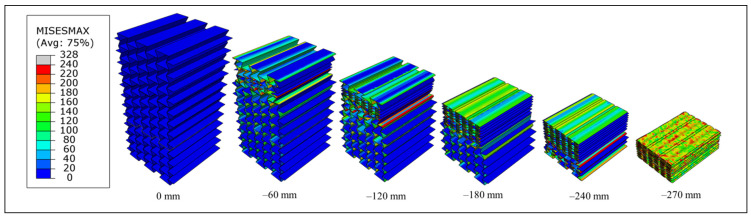
The progressive collapse and the corresponding stress values (in MPa) in the auxetic cores of the UGAD.

The progressive collapse and the corresponding stress values in the auxetic cores of the UGAD are shown in Figure 10. The stress values range mainly between 0 and 240 MPa (the ultimate strength of the aluminium alloy; Table 2). This indicates that the structure deforms mainly below the ultimate strength of the base material. Stress values from 240 MPa up to 328 MPa were only in the densification zone (at 270 mm of applied displacement), as illustrated in Figure 10. 

**Figure 11:**
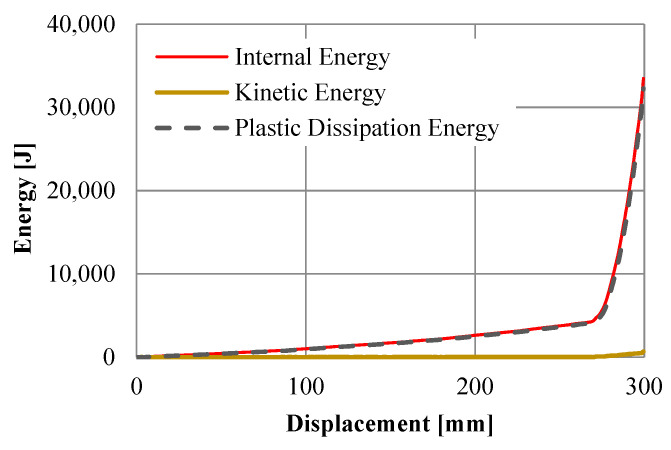
The energies observed in the numerical model of the UGAD.

**Figure 12:**
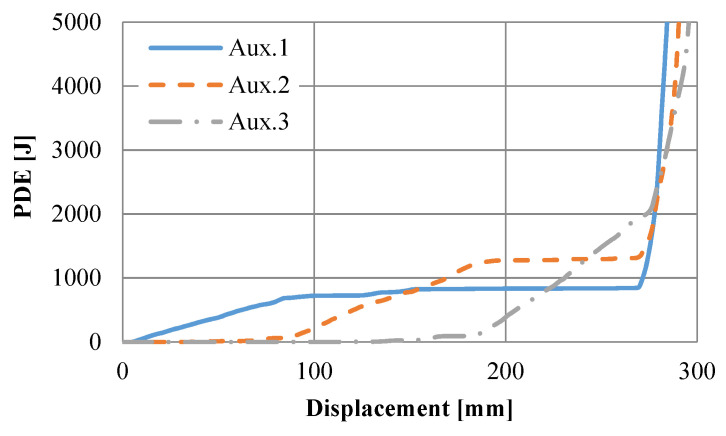
The plastic dissipation energy observed in each auxetic core of the UGAD.

The rise in internal energy was recorded during computer simulations to observe the amount of absorbed strain energy of the UGAD due to plastic deformation. Results (Figure 11) revealed that the internal energy in the auxetic cores is mainly composed of plastic dissipation energy (PDE) and negligible kinetic energy. This confirms the suitability of using the explicit solver for quasi-static analysis. Figure 12 shows the PDE observed in each auxetic core of the UGAD, where the summation of PDE values is equal to the total PDE (Figure 11). Moreover, within the plateau region (Figure 12), the maximum PDE observed in each auxetic core (Aux.1, Aux.2, and Aux.3) was in ascending order (828 J, 1277 J, and 2000 J), respectively. This was relative to the increase in (t) value, which reflects that the ‘graded’ UGAD system is cost-effective and chosen wisely.

The specific energy absorption (SEA), which is the amount of energy absorbed per unit mass of crushed material, can also be calculated for the fabricated UGAD and the representative computational model. The amount of energy absorbed is the area under the load–displacement curves in Figure 9b. Using the trapezoidal rule, the amount of energy absorbed within the plateau region (<250 mm of displacement) was 4575 J and 3863 J, for the experimental and numerical results, respectively. While the mass of the three auxetic cores altogether was 4.06 kg (Table 1), the SEA values within the plateau region (<250 mm of displacement) were 1126.8 J/kg and 951.6 J/kg, for the experimental and numerical results, respectively. This slight difference was expected and can be seen in Figure 9b, as the fabricated UGAD may not behave like the ideal computational model. The relatively high SEA values revealed the superior energy-absorption potential of the proposed cost-effective UGAD.

## 5. Conclusions

The previously published and patented idea of Uniaxial Graded Auxetic Damper (UGAD) has shown to be a promising energy absorbing solution for different applications. Hence, the first objective of this research was fabricating the UGAD using a relatively non-expensive technique rather than 3D printing. This cost-effectiveness may increase the production rate for different structural applications. The second objective was to test three different manufactured UGAD samples experimentally under quasi-static loading. The third objective was to build and validate a nonlinear finite element model that could be used in different applications without repeating the experimental tests. The research also covered material testing and proposed a simplified material model of the aluminium alloy (AW-5754, T-111) used in future computational simulations. The following conclusions can be summarised from the conducted study:

- The deformation patterns and progressive collapse of fabricated auxetic cores are in excellent agreement with the computational predictions, which validates the accuracy of the used computational and material models.
- The epoxy adhesive, *LOCTITE^®^ EA 9466*, was successfully used to bond the auxetic layers with high efficiency without needing extra rivets.
- A negative Poisson’s ratio (auxetic behaviour) was observed in all three re-entrant auxetic cores. The lateral shrinkage makes it easier to replace the deformed cores once crashed, without the need to replace the entire UGAD.
- The force–displacement relationships of the UGADs revealed a short linear response, followed by a broad plateau region, and a final rapid densification zone. The plateau region was divided into step-wise zones based on the behaviour of the three different auxetic cores. The fabricated samples showed high compatibility with the computational predictions.
- The computational model showed that the internal energy in the auxetic cores was mainly composed of plastic dissipation energy (PDE). Moreover, a relatively high value of specific energy absorption (SEA) was observed, which reflects the superior energy-absorption potential of the fabricated UGAD.

## 6. Patents

This core idea of this research work was recently patented by the patent office of the Republic of Poland [40], namely, “Tłumik jednoosiowy dla układów bezpieczeństwa bram, drzwi lub okien”/“Uniaxial damper as a safety system for gates, doors or windows”, patent number 238840. 

## Figures and Tables

**Table 1:** The geometrical properties of the three fabricated auxetic cores used in the proposed UGAD.

	Aux.1	Aux.2	Aux.3
Constant parameters	L=17.3 mm, cell angle θ = 60°		
*t* (mm)	0.8	1.0	1.2
t/L	0.046	0.058	0.069
Mass (kg)	1.109	1.348	1.603
Density ρ (kg/m^3^)	255.92	311.17	370.04
$\mathrm{Relative}\;\mathrm{Density}\;\rho^{*}$	0.10	0.12	0.14
Porosity p (%)	90.4	88.3	86.1
No. of fabricated samples	3	3	3

**Table 2:** The material parameters of the aluminium alloy (AW-5754, T-111) and its stress–strain key points.

Mechanical Properties	E (MPa)	ʋ	Density (kg/m^3^)
	63,177	0.33	2660
	Stress	Strain	Plastic Strain
Yield Point	172.09	0.0030	0.0000
	199.36	0.0200	0.0170
	219.08	0.0400	0.0370
	229.89	0.0600	0.0570
	235.01	0.0800	0.0770
Ultimate Point	239.81	0.1086	0.1056

## Data Availability

Data available on request due to restrictions. The data presented in this study are available on request from the corresponding author. The data are not publicly available due to grant and patenting restrictions.
